# Dementia and cognitive decline in Black Brazilians: a narrative review

**DOI:** 10.3389/fnhum.2026.1788136

**Published:** 2026-04-22

**Authors:** Natália Rocha Tardelli, Georgia Garcia, Marcio Luiz Figueredo Balthazar

**Affiliations:** 1 Geriatrics Division, Internal Medicine Department, Botucatu Medical School, São Paulo State University (UNESP), Botucatu, Brazil; 2 Department of Neurology, School of Medical Sciences (FCM), Universidade Estadual de Campinas (UNICAMP), São Paulo, Brazil

**Keywords:** African continental ancestry group, Black people, Brazil, cognitive aging, cognitive decline, dementia

## Abstract

**Background:**

Dementia prevalence is projected to rise most sharply in low-and middle-income countries, including Brazil. The Brazilian Black population (including individuals identified as Black and Brown) represents 56.5% of the population and is expected to comprise most older adults in the coming decades.

**Aims:**

This narrative review aims to synthesize studies on dementia, cognitive decline, and cognitive aging among Black Brazilians, analyzing publication characteristics and key findings to identify knowledge gaps and propose directions for future research.

**Methods:**

We searched PubMed, LILACS, and SciELO databases, and the SciSpace AI-powered tool. Eligible studies included those that: (a) examined the Brazilian Black population; and (b) provided descriptions or analyses of characteristics, clinical manifestations, risk factors, or responses to interventions related to cognitive decline, cognitive aging, and dementia. Publications that were not full-length articles were excluded.

**Results:**

We identified 18 papers (2000–2025), mostly cross-sectional, published in international journals. The median proportion of Black participants among samples was 39.5%. Seven studies were conducted in the state of São Paulo, and ten were carried out in the Southeast region of Brazil. A recurring sociodemographic feature in nine studies was the low educational attainment among Black participants. Twelve studies identified modifiable risk factors for dementia among black individuals, mainly related to socioeconomic disadvantages. Cognitive performance was assessed using various standardized instruments, such as the Mini-Mental State Examination and Clinical Dementia Rating Scale. We did not find studies about dementia care or any clinical trials.

**Conclusion:**

Our findings indicate the underrepresentation of Black individuals in dementia research. Ensuring the inclusion of Black populations in research requires investment in recruiting Black professionals into research teams, conducting interventions, and developing partnerships within Black communities. Existing Brazilian evidence suggests socioeconomic factors exert greater influence on cognitive function than genetic factors, underscoring the need for public policies that address social, income, healthcare access, and educational inequities. Beyond social investments, local research should develop culturally appropriate cognitive assessment tools and culturally compatible protective activities and lifestyles among marginalized populations. Finally, culturally tailored strategies for person-centered dementia care and carers’ support are needed.

## Introduction

Dementia is a chronic, incurable condition whose global prevalence has been steadily increasing (Nichols et al., 2022; Hao and Chen, 2025). Projections indicate that this rise will be most pronounced in low- and middle-income countries (Livingston et al., 2017; Nichols et al., 2022), such as Brazil. A Brazilian study published in January 2025 (Aliberti et al., 2025) estimated, using the Delphi technique, a dementia prevalence of 8.5% among individuals aged 60 years or older. By comparing estimates of dementia cases between 2018 and 2019, approximately 97,000 new cases were projected nationwide, equivalent to one new case every five minutes (Aliberti et al., 2025). Global projections (Nichols et al., 2022) indicate that the number of Brazilians living with Alzheimer’s disease and other dementias will triple by 2050, reaching an estimated 6.7 million people (an increase of more than 200%).

Moreover, in Brazil, aging is marked by profound socioeconomic, ethnoracial, and geographic inequalities (Szwarcwald et al., 2016, 2022). Evidence indicates that cumulative stress arising from social inequities, along with disparities in social and physical environments, directly influences dementia risk among historically marginalized and socially disadvantaged racial and ethnic groups (Alzheimer’s Association, 2025). In addition, longstanding structural inequities contribute to racial and ethnic disparities across multiple health outcomes, including the higher prevalence of chronic conditions associated with increased dementia risk, such as cardiovascular disease (Reddy et al., 2022, 2023; Mentias et al., 2023) and diabetes (Egede et al., 2024). These factors help explain the higher prevalence of Alzheimer’s disease and other dementias observed among non-Hispanic Black and Hispanic older adults in the United States (U.S.) (Anderson, 2004; Dilworth-Anderson et al., 2008; Steenland et al., 2016; Power et al., 2021). U.S. data indicates 19% of Black and 14% of Hispanic adults age 65 and older have Alzheimer’s dementia compared with 10% of White older adults (Rajan et al., 2021); even these populations are probably underdiagnosed (Matthews et al., 2019). Consistent with these findings, most other prevalence studies indicate that Black older adults are approximately twice as likely to have Alzheimer’s or other dementias as White older adults (Gurland et al., 1999; Potter et al., 2009; Rajan et al., 2019; Manly et al., 2022).

In Brazil, no reliable estimates of dementia prevalence by ethnicity are available, as existing population-based data cannot be extrapolated to the national level (Brasil, 2025). However, sociodemographic data indicate that Black and Brown (“Pardo”) populations are disproportionately affected by limited access to education (Marteleto, 2012; IBGE, 2024) and healthcare (Li et al., 2021; Medeiros et al., 2023; Miranda and Lima, 2023; Coelho et al., 2025; Ferezin et al., 2025), as well as higher levels of poverty, (De Macedo, 2020; IBGE, 2024) food insecurity (Gubert et al., 2017; IBGE, 2024; Luiz et al., 2025; Ribeiro et al., 2025), and illiteracy (IBGE, 2024). All of these factors are known to increase the risk of developing dementia. In this review, individuals identified as Black and Brown (“Pardo”) were grouped under the category “Black,” as both populations experience similar forms of racism and share comparable adverse health outcomes (Travassos and Williams, 2004; Suemoto et al., 2025).

Currently, Black (and Brown) individuals represent 56.5% of the Brazilian population (IBGE, 2024), corresponding to more than 121 million people, and account for over 70% of those living in poverty and extreme poverty in the country (IBGE, 2024). They also continue to experience disproportionately limited access to formal education (IBGE, 2024). This is particularly concerning given that demographic projections suggest Black individuals may constitute the majority of older adults in Brazil in some decades, due to their growing number and proportion (Tramujas Vasconcellos Neumann and Albert, 2018; Plácido et al., 2023; IBGE, 2024).

Limited access to formal education contributes to reduced cognitive reserve (Suemoto et al., 2022, 2025) in a population that already faces lower health literacy and restricted access to healthcare services. These factors may increase cerebral vulnerability and the risk of cognitive decline (Meng and D’Arcy, 2012), accelerating or anticipating cognitive aging (Feter et al., 2025), which should represent a critical public health concern.

Despite this urgent need, Black individuals and other vulnerable populations remain underrepresented in existing studies on dementia (Krishnan et al., 2024; Arellanes et al., 2024; Feter et al., 2025), which constitutes a major limitation in the literature and hinders understanding of clinical manifestations, prevention strategies, care approaches, and treatment responses among Black populations living with neurodegenerative diseases and other dementias.

Considering Brazil’s demographic composition (IBGE, 2024) and its ongoing demographic and epidemiological transition (Borges, 2017), this narrative review aims to identify studies published to date on dementia, cognitive decline, and cognitive aging among Black individuals, including Black and Brown populations, in Brazil. Through the analysis of selected articles, we seek to describe the main journals in which these studies appear, the pattern of publication output over time, and the settings in which the research was conducted. We also aim to examine the proportion of Black individuals within sample populations, outline key sociodemographic characteristics of these participants, and analyze the methods used to assess cognitive performance among Black Brazilians. Additionally, we intend to identify existing cohorts and clinical trials, classify studies according to their primary themes, and evaluate the risk factors for dementia in this population, including whether these factors are modifiable. Based on these findings, we aim to highlight existing knowledge gaps and provide a forward-looking analysis to guide future research.

## Materials and methods

**Figure 1:**
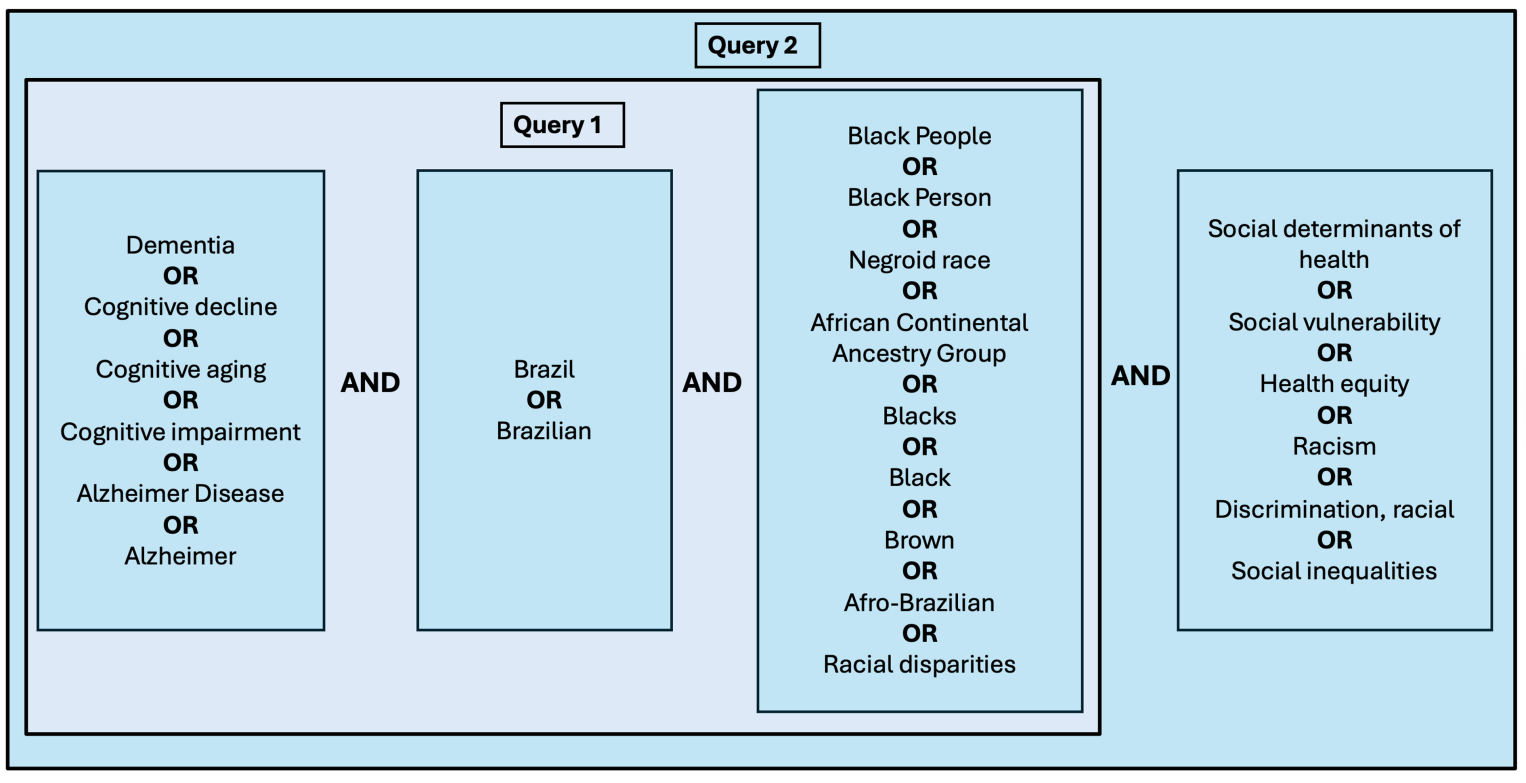
Search Queries. Two structured search queries were utilized. Query 1 included the terms (Dementia OR Cognitive decline OR Cognitive aging OR Cognitive impairment OR Alzheimer disease OR Alzheimer) AND (Brazil OR Brazilian) AND (Black People OR Black Person OR Negroid race OR African Continental Ancestry Group OR Blacks OR Black OR Brown OR Afro-Brazilian OR Racial disparities). Query 2 encompassed all terms from Query 1 and additionally incorporated the domain (Social determinants of health OR Social vulnerability OR Health equity OR Racism OR Discrimination, racial OR Social inequalities). Boolean operators (AND/OR) were used to combine terms across domains.

Searches were conducted in the PubMed, LILACS, and SciELO databases using the search strategy described in Figure 1 and Supplementary Material 1. In addition, the SciSpace platform was used to complement the database searches, applying the same queries described in Figure 1 and Supplementary Material 1.

All studies identified through these sources underwent the same screening, eligibility assessment, and data extraction procedures. The eligibility criteria for study selection were as follows: (a) inclusion of the Brazilian Black population as one of the study populations; and (b) provided descriptions and/or analyses of characteristics, clinical manifestations, risk factors, or responses to interventions related to cognitive decline, cognitive aging, and dementia.

The exclusion criteria were as follows: (a) publications that were not full-length articles published in indexed journals (such as conference abstracts or preprints); (b) papers that did not address dementia, cognitive decline, cognitive aging, or cognitive impairment; and (c) papers that examined Black populations from countries other than Brazil, rather than among Brazilian Black individuals. No restrictions were applied regarding publication date or language. The papers were selected by two independent researchers between November 10, 2025, and December 4, 2025. In the first stage, articles were screened based on their titles. In the second stage, abstracts were reviewed, and studies were included or excluded according to the eligibility criteria described above. Data extraction was conducted by one of the investigators, who independently reviewed each paper in full. At this stage, studies that did not meet the eligibility criteria, despite having been selected in the previous steps, were excluded from the review.

During data collection and analysis, the selected papers were organized according to study topic, author, year of publication, and study design. For each paper, the following elements were also examined and described: study setting, time frame, characteristics of the study population, whether the study identified risk factors for dementia, and whether the identified risk factors were modifiable.

## Results

### Article selection

**Figure 2:**
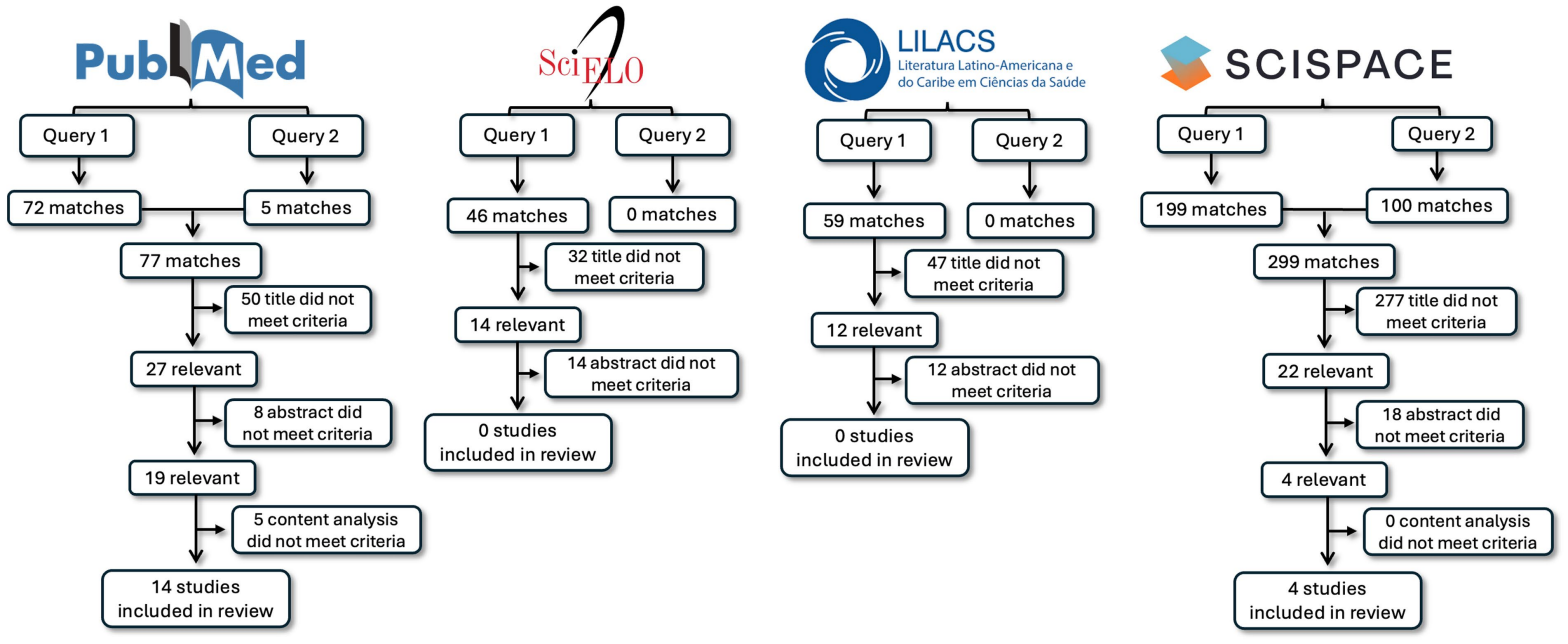
Flow diagram of the literature search, screening, and inclusion process. This figure presents the identification and selection of studies retrieved from PubMed, SciELO, and LILACS databases, as well as from SciSpace AI-powered research. For PubMed, SciELO, and LILACS, two independent search queries were conducted in each database, and records were screened by title, abstract, and full-text content according to predefined eligibility criteria. SciSpace search results, obtained using two search queries, are presented separately and were screened using the same eligibility criteria. The final number of studies included in the review is reported for each source.

**Figure 3:**
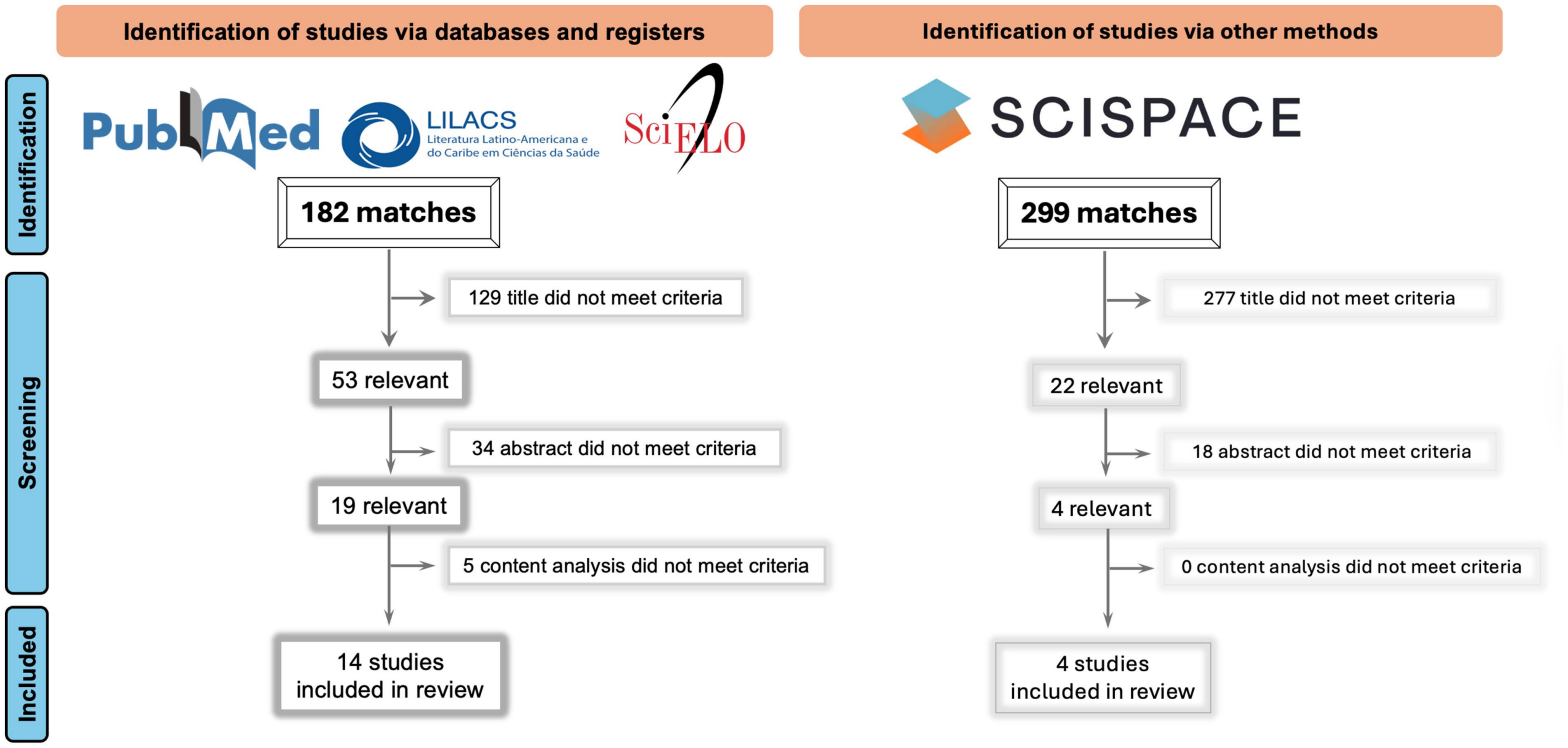
Study identification and selection process. This figure illustrates the identification of studies via bibliographic databases (PubMed, SciELO, and LILACS) and via other methods (SciSpace). Screening was performed at the title, abstract, and full-text levels based on predefined eligibility criteria, resulting in the final set of studies included in the review.

Figures 2, 3 schematically illustrate the article selection process. Searches of the PubMed, LILACS, and SciELO databases yielded a total of 183 publications. After screening titles, abstracts, and full texts, 14 papers were selected, all of which were retrieved from PubMed. The search conducted on the SciSpace website identified 299 publications, of which four were included after title, abstract, and full-text review. Overall, the search process resulted in 18 papers, all of which were full-length articles published in indexed journals and met the eligibility criteria described in the Methods section.

### Reviews

**Figure 4:**
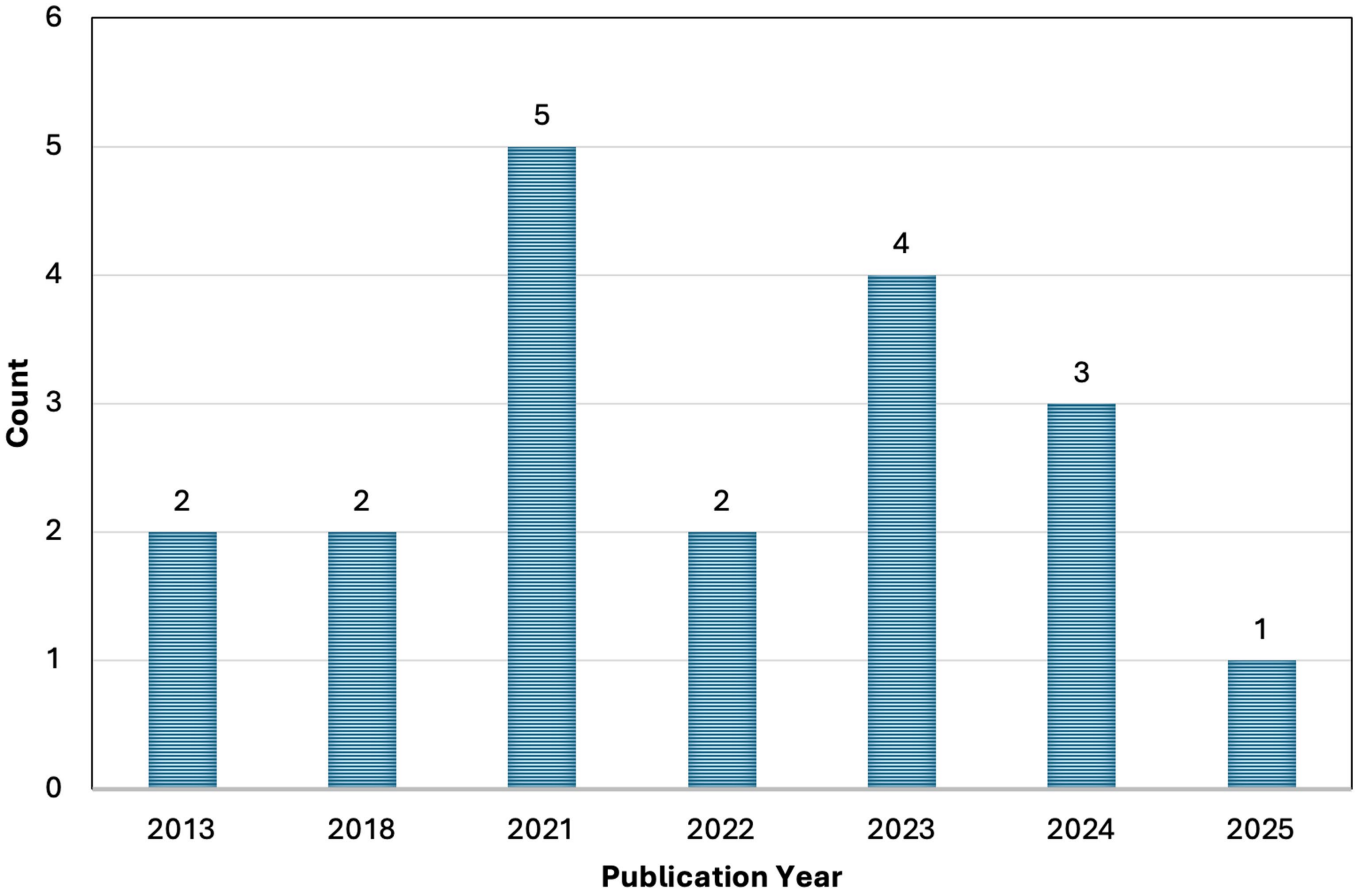
Annual publication output. Number of articles included in the review by year of publication.

Table 1 summarizes the characteristics and key findings of the included studies. All studies were published between 2000 and 2025, with 15 of the 18 studies published between 2021 and 2025, including five articles published in 2021 alone (Figure 4). Most of the selected articles were published in international journals; two were published in Brazilian journals, and 16 in international periodicals.

**Table 1:** Characteristics and main findings of included studies.

Theme	Author	Publication year	Journal	Reference	Study design	Setting	Time frame	Population (*n*)	Black population^a^ (*n* and %)	Outcome	Dementia risk factor studied	Is it a protective or risk factor for dementia?	Is it a modifiable risk factor?
African ancestry and Alzheimer’s disease	de-Andrade et al.	2000	*Braz J Med Biol Res.*	De-Andrade et al. (2000)	Cross-sectional	Porto Alegre, Brazil	Not specified	546 participants	100 participants (18%)	Presence of APOE ε4	Presence of APOE ε4 gene polymorphism	Risk factor	No
	Naslavsky et al.	2022	*Mol Psychiatry.*	Naslavsky et al. (2022)	Cohort	São Paulo, Brazil	2004–2017	400 participants	120 participants (30%)	Presence of AD-related neuropathology and APOE ε4 gene polymorphism	Genetic African Ancestry and APOE ε4	Protective factor	No
	Schlesinger et al.	2013	*Mol Psychiatry*	Schlesinger et al. (2013)	Cross-sectional	São Paulo, Brazil	2004–2009	202 brain samples	112 brain samples (55.4%)	Presence of AD-related neuropathology	Genetic African Ancestry	Protective factor	No
	Bicalho et al.	2013	*Int J Geriatr Psychiatry*	Bicalho et al. (2013)	Cross-sectional	Minas Gerais, Brazil	2006–2009	266 participants	54 participants (39.5%)	Presence of APOE ε4 gene polymorphism	Genetic African Ancestry and social and clinical factors	Risk factor	Yes – social and clinical factors No – Genetic Factors
African ancestry and cognitive decline	Lima-Costa et al.	2018	*J Am Geriatr Soc.*	Lima-Costa et al. (2018)	Cohort	Bambui, Minas Gerais, Brazil	January 1997–December 2011	1,215 participants	236 participants^b^ (19.5%)	Cognitive Decline	African Ancestry	Not a risk factor, nor a protective factor	No
Discrimination and dementia risk	Farfel et al.	2021	*Alzheimers Dement*	Farfel et al. (2021)	Cross-sectional	São Paulo, Brazil – PARDoS	2020	899 participants	388 participants (34.3%)	Presence of dementia and cognitive impairment	Discrimination	Risk factor	Yes
Literacy as a protective factor	Capuano et al.	2021	*J Alzheimers Dis.*	Capuano et al. (2021b)	Cross-sectional	São Paulo, Brazil – PARDoS	2020	1,818 participants	617 participants (34%)	Dementia	Literacy (verbal, numeracy and music)	Protective factor	Yes
	de Paula França Resende et al.	2024	*Alzheimers Dement*	(De Paula França Resende et al., 2024)	Cross-sectional	Belo Horizonte, Minas Gerais, Brazil	February–July, 2019	35 participants	28 participants (80%)	Enhanced hippocampal connectivity	Healthy Literacy	Protective factor	Yes
Neuroticism, negative life events and dementia	Capuano et al.	2021	*Int J Geriatr Psychiatry.*	Capuano et al. (2021a)	Cross-sectional	São Paulo, Brazil – PARDoS	2016–2020	1,747 participants	584 participants (33.5%)	Dementia	Neuroticism and negative life events	Risk factor	Yes
Purpose in life and dementia	Wilson et al.	2021	*J Int Neuropsychol Soc.*	Wilson et al. (2022)	Cross-sectional	São Paulo, Brazil – PARDoS	Not Specified	1,514 participants	510 participants (33.7%)	Dementia	Purpose in life	Protective factor	Yes
Race and modifiable risk factors for dementia	Borelli et al.	2023	*Alzheimers Dement*	Borelli et al. (2023)	Cross-sectional	ELSI-Brazil^c^	2015-2016	9,070 participants	5,164 participants (57%)	Not applied^d^	Ten modifiable risk factors for dementia^d^	Risk factor	Yes
	Suemoto et al.	2023	*Alzheimers Dement.*	Suemoto et al. (2023)	Cross-sectional	ELSI-Brazil	2015–2016	9,412 participants	5,170 participants (55%)	Not applied^e^	Twelve dementia risk factors^e^	Risk factor	Yes
	Simon et al.	2023	*Alzheimers Dement*	Simon et al. (2023)	Perspective Review	Literature generated by the United States and Brazilian governments	Not specified	–	–	–	Social, economic, literacy, and health factors.	Risk factor	Yes
Racial inequities in cognitive decline	Feter et al.	2025	*Neurology*	Feter et al. (2025)	Cohort	ELSA-Brazil^f^	2008–2019	10,308 participants	4,412 participants (43%)	Cognitive Decline	Societal Inequities	Risk factor	Yes
	Plácido et al.	2023	*Rev Saúde Pública.*	Plácido et al. (2023)	Cross-sectional	ELSI-Brazil	2015–2016	8,760 participants	5,170 participants (59%)	Intrinsic Capacity^f^	Socioeconomic disadvantages, illiteracy, and poor self-rated health	Risk Factor	Yes
Social and emotional isolation and dementia	Wilson et al.	2024	*Int Psychogeriatr.*	Wilson et al. (2024)	Cross-sectional	São Paulo, Brazil – PARDoS	2020	1,493 participants	509 participants (34.1%)	Dementia	Social and emotional isolation	Risk factor	Yes
Social participation and cognitive health	Simon et al.	2024	*Int Psychogeriatr.*	Simon et al. (2025)	Cross-sectional	Quilombola Communities, Bequimão, Maranhão, Brazil.	2018–2020	221 participants	195 participants (88.3%)	Cognitive impairment and dementia	Social participation	Protective factor	Yes
Subjective cognitive decline and race	Borelli et al.	2022	*Alzheimers Dement*	Borelli et al. (2022)	Cross-sectional	ELSI-Brazil	2015–2016	6,887 participants	3,699 participants (53%)	Subjective cognitive decline	Subjective cognitive decline	Risk factor	Mostly yes -many subjective cognitive declines exist due to modifiable risk factors

a The term Black population refers to individuals who self-identify identify as Black or Brown (mixed race).

b Considering the Black population, participants with high Genomic African Ancestry.

c The ELSI-Brazil cohort is a nationally representative, home-based longitudinal survey of adults aged 50 years and older, conducted in 70 municipalities across Brazil’s five geographic regions (North, Northeast, Midwest, Southeast, and South).

d Borelli et al. estimated the prevalence of ten modifiable dementia risk factors among Brazilian older adults: lower educational attainment, hearing loss, hypertension, alcohol abuse, obesity, smoking, depression, social isolation, physical inactivity, and diabetes.

e Suemoto et al. study calculated the population attributable fraction for 12 dementia risk factors and assessed variation by race and socioeconomic characteristics across Brazil’s macro-regions.

f Data from ELSA-Brazil were collected in public universities located in 6 Brazilian state capitals: the South (Porto Alegre), Southeast (Belo Horizonte, Vitória, and Rio de Janeiro), and Northeast (Salvador) regions.

^g^Plácido et al. explored associations among race/color, gender, and intrinsic capacity (a construct encompassing cognitive health) among Brazilian older adults.

AD, Alzheimer’s Disease; PARDoS, Pathology, Alzheimer’s and Related Dementia Study; ELSI-Brazil, Brazilian Longitudinal Study of Aging; ELSA-Brazil, Brazilian Longitudinal Study of Adult Health.

Of the 18 studies, 14 were cross-sectional (De-Andrade et al., 2000; Bicalho et al., 2013; Schlesinger et al., 2013; Capuano et al., 2021a,b; Farfel et al., 2021; Borelli et al., 2022, 2023; Wilson et al., 2022, 2024; Plácido et al., 2023; Suemoto et al., 2023; De Paula França Resende et al., 2024; Simon et al., 2025), three were cohort studies (Lima-Costa et al., 2018; Naslavsky et al., 2022; Feter et al., 2025) and one was a perspective review (Simon et al., 2023). The proportion of Black participants (including Black and Brown individuals) ranged from 18 to 88.3%. The median rate among samples was 39.5%, and the average rate was 45.14%. A common sociodemographic characteristic in 9 of the 18 sample studies was the low level of educational attainment among the Black participants (Lima-Costa et al., 2018; Capuano et al., 2021b; Borelli et al., 2022; Wilson et al., 2022; Plácido et al., 2023; Suemoto et al., 2023; De Paula França Resende et al., 2024; Feter et al., 2025; Simon et al., 2025). Twelve (Bicalho et al., 2013; Lima-Costa et al., 2018; Capuano et al., 2021b; Farfel et al., 2021; Borelli et al., 2022, 2023; Plácido et al., 2023; Suemoto et al., 2023; De Paula França Resende et al., 2024; Feter et al., 2025; Simon et al., 2025) of the 18 studies in our review identified modifiable risk factors for dementia among black individuals.

Cognitive performance was assessed using different instruments across studies. The Clinical Dementia Rating Scale and Informant Questionnaire on Cognitive Decline in the Elderly were applied in the Pathology, Alzheimer’s and Related Dementia Study (PARDoS) (Capuano et al., 2021a,b; Farfel et al., 2021; Wilson et al., 2022, 2024). The Mini-Mental State Examination in Lima-Costa et al. (2018) and Simon et al. (2025) studies. Feter et al. (2025) employed the Consortium to Establish a Registry for Alzheimer’s Disease, Verbal fluency tests (animals and letter F) and the Trail-making test (version B). Plácido et al. (2023) used the Verbal Fluency Test.

Seven studies were conducted in the state of São Paulo (Schlesinger et al., 2013; Capuano et al., 2021a,b; Farfel et al., 2021; Naslavsky et al., 2022; Wilson et al., 2022, 2024) and ten were carried out in the Southeast region of Brazil (Bicalho et al., 2013; Schlesinger et al., 2013; Lima-Costa et al., 2018; Capuano et al., 2021a,b; Farfel et al., 2021; Naslavsky et al., 2022; Wilson et al., 2022, 2024; De Paula França Resende et al., 2024). Five studies (Capuano et al., 2021a,b; Farfel et al., 2021; Wilson et al., 2022, 2024) used data from the Pathology, Alzheimer’s and Related Dementia Study (PARDoS), four studies (Borelli et al., 2022, 2023; Plácido et al., 2023; Suemoto et al., 2023) analyzed data from the Brazilian Longitudinal Study of Aging (ELSI-Brazil), and one study (Feter et al., 2025) was based on data from the Brazilian Longitudinal Study of Adult Health (ELSA-Brazil).

PARDoS is a community-based study of aging and dementia that prioritized enrollment of Black or mixed-race individuals aged 65 years or older at death, as well as White decedents with eight or fewer years of education (Farfel et al., 2021). The ELSI-Brazil cohort is a nationally representative, home-based longitudinal survey of adults aged 50 years and older, conducted in 70 municipalities across Brazil’s five geographic regions (North, Northeast, Midwest, Southeast, and South) (Lima-Costa et al., 2023). Finally, data from the ELSA-Brazil were collected in public universities located in six Brazilian state capitals: Porto Alegre (South), Belo Horizonte, Vitória, and Rio de Janeiro (Southeast), and Salvador (Northeast) (Feter et al., 2025).

The studies addressed a broad range of topics, which were organized and summarized into thematic categories, as in Table 1. Four articles were classified under the theme “African Ancestry and Alzheimer’s Disease” because they examined the influence of genetic African ancestry on Alzheimer’s neuropathology (Schlesinger et al., 2013; Naslavsky et al., 2022) and APOE gene polymorphism/genotype (Bicalho et al., 2013; Naslavsky et al., 2022) among Black participants. Similarly, De-Andrade et al. (2000) analyzed APOE gene polymorphisms among self-declared Black individuals without the genetic ancestry information. Beyond Alzheimer’s Disease neuropathology and APOE genotype, Lima-Costa et al. (2018) investigated whether African genetic ancestry influences the rate of cognitive decline among 1,215 Brazilian older adults over a 15-year period.

In addition to genetic factors, several studies examined social determinants of cognitive health and dementia. Ten studies analyzed social, demographic, and economic factors in relation to cognitive decline and dementia among the Brazilian Black population (Suemoto et al., 2023; Farfel et al., 2021; Capuano et al., 2021b; De Paula França Resende et al., 2024; Borelli et al., 2023; Simon et al., 2023; Feter et al., 2025; Plácido et al., 2023; Wilson et al., 2024; Simon et al., 2025). For example, Farfel et al. (2021) investigated the impact of lifetime experiences of discrimination on the risk of dementia and cognitive impairment among 899 older Brazilians aged 65 years or older, 34.3% of whom were Black. Capuano et al. (2021b) examined the association between verbal, numeracy, and musical litteracy and dementia among 1,818 older Brazilians, including 617 Black participants. De Paula França Resende et al. (2024) investigated the association between hippocampal connectivity and health literacy among 35 illiterate individuals, 28 of whom were Black.

Similarly, two studies (Suemoto et al., 2023; Borelli et al., 2023) addressed social and health-related factors by examining modifiable risk factors for dementia in a sample of 9,070 Brazilian older adults from the ELSI-Brazil cohort. Borelli et al. (2023) estimated the prevalence of ten modifiable dementia risk factors across five racial groups (White, Black, Brown, Asian, and Indigenous). Suemoto et al. (2023) calculated the population attributable fraction for 12 dementia risk factors and assessed variation by race and socioeconomic characteristics across Brazil’s macro-regions.

In line with these concerns, Simon et al. (2023) conducted a perspective review on mental and brain health, including Alzheimer’s disease and related dementias, among native Brazilians and Brazilian immigrants in the United States. Their findings were organized into major thematic areas such as Brazilian identity and ancestry, sociocultural factors, and social inequities in Brazil. Particular emphasis was placed on dementia risk factors associated with socioeconomic disparities among Black and Brown Brazilian populations.

One topic addressed by Feter et al. (2025) and Plácido et al. (2023) concerns racial inequities in cognitive decline. Feter et al. (2025) conducted a cohort study with 10,308 participants from the ELSA-Brazil study, including 1,510 Black individuals and 2,902 Brown (mixed-race) individuals, to examine cognitive function trajectories over approximately nine years across racial and ethnic groups. Plácido et al. (2023) explored associations among race/color, gender, and intrinsic capacity (a construct encompassing cognitive health) among Brazilian older adults, 59% of whom were Black.

Regarding social participation and cognitive health, Simon et al. (2025) examined the influence of social participation on cognitive health among older adults in rural Quilombola communities in Maranhão, Brazil. This cross-sectional study included 221 older adults aged 60 to 104 years from 11 underserved Quilombola communities. Additionally, Wilson et al. (2024) investigated associations between social and emotional isolation and dementia risk among 1,493 older Brazilians, 34.1% of whom were identified as Black by their descendants.

Beyond ancestry and social aspects, other studies examined psychosocial aspects and their influence on dementia. Capuano et al. (2021a) examined the associations among neuroticism, negative life events and dementia using data collected through legal representatives in the PARDoS study. Wilson et al. (2022) tested the hypothesis that a higher level of purpose in life is associated with a lower likelihood of dementia and mild cognitive impairment among older Brazilians, 33.7% of whom were Black.

Finally, Borelli et al. (2022) investigated the prevalence of subjective cognitive decline in Brazil and its associated risk factors, comparing prevalence across racial groups, including Black (9.95%), Brown (48.4%), and White individuals.

## Discussion

Brazil is a continental country characterized by vast territorial extent and marked socioeconomic inequalities (Azzoni and Haddad, 2018; Pochmann and Da Silva, 2020; IBGE, 2024). Our findings indicate a small number of publications on dementia among Black individuals in Brazil, most of which were published recently, and a predominance of study populations from Southeastern Brazil (the wealthiest region of the country) (Azzoni and Haddad, 2018). Together, these findings reinforce an exclusionary pattern within dementia research. Despite the highest poverty rates and largest proportion of Black people being concentrated in the Northeast region (IBGE, 2024), followed by the North; these populations remain underrepresented in dementia research. More broadly, studies on dementia and cognitive aging reflect long-standing structural inequalities, as Black populations are frequently excluded from large cohort studies (Krishnan et al., 2024) and clinical trials (Gilmore-Bykovskyi et al., 2019; Brijnath et al., 2022; Mitchell et al., 2024).

A relevant finding is that three studies (Bicalho et al., 2013; Schlesinger et al., 2013; Naslavsky et al., 2022) demonstrated that African genetic ancestry is not associated with Alzheimer’s disease pathology or with genetic factors that increase the risk of developing the disease. Additionally, Lima-Costa et al. (2018) found that genetic ancestry did not predict the progression of memory loss with aging, concluding that the pace of brain aging is similar regardless of ancestral origin.

Existing Brazilian evidence on the etiologies of cognitive decline among Black and White individuals (Bicalho et al., 2013; Schlesinger et al., 2013; Lima-Costa et al., 2018; Naslavsky et al., 2022; Borelli et al., 2023; Suemoto et al., 2024) indicates that socioeconomic factors exert a greater influence on cognitive function than hereditary factors. These findings are corroborated by international literature, showing that the higher prevalence of Alzheimer’s Disease and other dementias among Black individuals (Mayeda et al., 2016; Power et al., 2021; Shiekh et al., 2021; Alzheimer’s Association, 2025) is not explained by genetic factors (Alzheimer’s Association, 2025). Instead, international research suggests that differences in life experiences, socioeconomic indicators, and health conditions most likely explain group differences in risk for Alzheimer’s Disease and other dementias.

Epidemiological studies by Borelli et al. (2023) and Suemoto et al. (2023), which examined the prevalence of modifiable risk factors for dementia in Brazil, further illuminate the socioeconomic influence on dementia risk among Black Brazilians. These studies found that Black and Brown participants were disproportionately affected by factors associated with socioeconomic inequality, including higher illiteracy rates and lower family income. Indeed, a common sociodemographic characteristic across many samples was the low level of educational attainment among Black participants (Lima-Costa et al., 2018; Capuano et al., 2021b; Borelli et al., 2022; Wilson et al., 2022; Plácido et al., 2023; Suemoto et al., 2023; De Paula França Resende et al., 2024; Feter et al., 2025; Simon et al., 2025). This pattern aligns with official national reports (IBGE, 2024) and recent literature (Suemoto et al., 2025), which indicate that the Black population faces structural disadvantages, including lower rates of secondary and higher education completion and, in low-income groups, illiteracy rates are up to six times higher than those observed in the wealthiest strata (IBGE, 2024).

It is well established that literacy is a cognitive reserve proxy (Meng and D’Arcy, 2012; Chapko et al., 2018; Song et al., 2022; Suemoto et al., 2022) therefore, lower literacy rates are associated with a greater risk of dementia and its earlier onset (Kaup et al., 2014; Arce Rentería et al., 2019), as demonstrated in Feter et al. (2025) study, where cognitive decline onset was nearly 10 years earlier in Black and Brown adults compared to White adults, and the proportion of individuals with less than elementary education was three times higher among Black adults and twice as high among Brown adults compared to White adults.

Moreover, it is known that longstanding structural inequities have resulted in limited access to healthcare services for Black populations compared to White population (Mayeda et al., 2016; Feter et al., 2021; Power et al., 2021; Trani et al., 2025), leading to poorer control of comorbidities such as systemic arterial hypertension (Petrea et al., 2020; Jansen et al., 2022), cardiovascular disease (Reddy et al., 2022, 2023; Mentias et al., 2023), and diabetes (Egede et al., 2024), thereby explaining a higher burden of vascular dementia among Black populations (Suemoto et al., 2024). This interpretation is further supported by Parodi et al. (2023), who evidenced that small vessel disease is more common among marginalized populations.

In line with this, Suemoto et al. (2025) demonstrated that the potential for dementia prevention in Brazil is nearly 60%, substantially higher than that observed in high-income countries, largely due to the high prevalence of modifiable risk factors associated with the limited access to education and healthcare services. For example, the population attributable fractions for dementia related to low educational attainment and depression in Brazil (Suemoto et al., 2025) exceed the global averages reported by the 2024 Lancet Commission (Livingston et al., 2017), underscoring how existing social and economic inequalities in the country directly influence access to education and health care. The evidence underscores the need for public policies that address social, income, healthcare access, and educational inequities in Brazil, to reduce dementia prevalence; thereby alleviating suffering among people living with dementia and their family members and lowering healthcare costs.

One important gap concerns the lack of studies investigating the impact of culturally appropriate lifestyle and activities of daily life on cognitive reserve among Black and other disadvantaged or marginalized populations. For example, the study by Simon et al. (2025) demonstrated that social engagement, particularly participation in religious activities, appeared to act as a protective factor, mitigating barriers such as high illiteracy rates and socioeconomic vulnerabilities among the Quilombola population. This finding raises the question of whether the absence of research on cognitive aging among vulnerable populations in low and middle-income countries has led us to import knowledge and models from high-income countries and wealthier populations without adequate cultural adaptation, thereby hindering both the interpretation of Brazilian studies’ results and the implementation of public policies. This was in line with the existing literature, where three systematic reviews on cognitive reserve proxies found that all existing studies were conducted in high-income countries (Meng and D’Arcy, 2012; Chapko et al., 2018; Song et al., 2022), with the exception of one Argentine cross-sectional study, which included a small sample of 33 participants (Song et al., 2022).

One of the most critical issues regarding the low investment in research aimed at developing culturally appropriate models for vulnerable populations in Brazil within the field of cognition is the cultural inadequacy of many cognitive and neuropsychological assessments for our population. In our findings, cognitive performance was assessed using different instruments across studies (Lima-Costa et al., 2018; Capuano et al., 2021a,b; Farfel et al., 2021; Wilson et al., 2022, 2024; Plácido et al., 2023; Feter et al., 2025; Simon et al., 2025). However, many of the cognitive tests applied in research and clinical practice in Brazil were validated for the population of São Paulo (Melo and Barbosa, 2015; Martins et al., 2019), which differs significantly in cultural, economic, and educational characteristics from those in other regions of the country.

In the study by Simon et al. (2025), the Mini-Mental State Examination (MMSE), Katz Index, and Lawton Scale were applied for dementia assessment. The median MMSE score was 18, lower than the cutoff values previously established for illiterate populations in the state of São Paulo (Bertolucci et al., 1994; Almeida, 1998; Brucki et al., 2003), raising concerns about potential misclassification of cognitive impairment. Although evidence supports the MMSE’s sensitivity and specificity for dementia screening in low-socioeconomic and minimally literate populations when education-specific cutoffs are applied (Brucki et al., 2003; Castro-Costa et al., 2009), studies from São Paulo (Scazufca et al., 2009) and the state of Rio de Janeiro (Laks et al., 2007) still report high misclassification rates among illiterate individuals, underscoring limitations for this subgroup.

Brazil’s cultural heterogeneity and the profound disparities in educational quality further challenge the generalizability of MMSE cutoffs across settings. Even the authors who validated cutoffs for low-socioeconomic status populations (Brucki et al., 2003) note that variability in primary education (e.g., number of school days, daily instructional hours, and teacher availability) produces heterogeneous response profiles, especially among those with very low schooling. This heterogeneity is evident in the literature even within groups classified as illiterate, and is far less pronounced at higher education levels. Another cognitive screening tool validated for Brazilian older adults is the Brazilian version of the Montreal cognitive assessment (Memória et al., 2013), which is a better option for detecting mild cognitive impairment in populations with at least 4 years of schooling; however, it has limited applicability for illiterate individuals.

Furthermore, in Simon et al. (2025), 82.2% of the study population had at least one or two difficulties on the Lawton Scale; however, some items in the Lawton Scale appear culturally inappropriate for the Quilombola community context (Santos et al., 2022; Censo 2022, 2025). For instance, the item “mode of transport” (drives independently on public transportation or drives own car) does not reflect the reality of Quilombola communities, which are predominantly located in rural and remote areas with limited infrastructure and services, including public transportation, and high poverty rates that hinder car ownership (Machado et al., 2020; IBGE, 2024; Censo 2022, 2025). Previous literature also reports greater difficulties among older Quilombolas in using transportation when applying the Lawton Scale (Santos et al., 2022), suggesting that these limitations may reflect structural barriers rather than functional decline. Supporting this interpretation, 56.5% of participants reported participation in organized social activities (Simon et al., 2025).

A first step toward advancing research and clinical practice in the field of cognition in low- and middle-income countries, including Brazil, may be investing in studies that develop culturally appropriate cognitive assessment tools for the diverse populations that comprise our country.

Another significant gap concerns the paucity of studies addressing the care of Black individuals living with dementia and their carers. In Brazil and across Latin America, such care is predominantly provided by family members (Ibáñez et al., 2021). Given that dementia is a chronic and incurable condition, one of the greatest benefits of its diagnosis is the ability to provide care that aligns with the values and preferences of the person living with dementia (Husebo et al., 2011; Livingston et al., 2017; Davies et al., 2024), as well as guidance and support for family members (Van Der Steen et al., 2024), thereby reducing the burden of suffering associated with the disease (Mitchell et al., 2009; Moens et al., 2014). Addressing this gap requires educating healthcare professionals at all levels of care in palliative care for people living with dementia and promoting research aimed at developing culturally appropriate care models. Evidence indicates that perceptions of autonomy (Tardelli et al., 2025), the role of the family (Tripodoro et al., 2024), and what constitutes a good death (Mikelyte et al., 2025) vary across cultures, suggesting that importing palliative care models from high-income countries may hinder effective implementation in low and middle-income countries, such as Brazil.

Furthermore, we did not find clinical trials, whether pharmacological or non-pharmacological, on Alzheimer’s disease or other dementias conducted in Brazil that included Black populations. In fact, the scarcity of nationally conducted clinical trials in the field of dementia (Barbosa et al., 2024; Ramos et al., 2024) limits our understanding of intervention responses in our population. Despite Black individuals being disproportionately impacted by Alzheimer’s (Alzheimer’s Association, 2025) they remain underrepresented in Alzheimer’s trials internationally (Faison et al., 2007; Shin and Doraiswamy, 2016; Manly and Glymour, 2021; Raman et al., 2021; Grill et al., 2024), which further hinders the extrapolation of study findings to Black Brazilians.

To ensure the inclusion of Black populations in future research, it seems necessary first to consider the substantial barriers that marginalized groups face in accessing health services (Constante and Bastos, 2021; Sato et al., 2026) and, even more critically, major research centers (Barreto et al., 2011). Marginalized populations, including Black communities, often reside far from research institutions, experience higher levels of poverty and informal employment (IBGE, 2024), being frequently unable to absorb the direct and indirect costs of participating in studies (for instance, missing work to attend research visits). Therefore, investment in conducting research interventions within the communities where these populations live appears to be a first step (Barreto et al., 2011).

Another essential step for recruiting Black Brazilians is the inclusion of Black researchers and professionals in research teams (Okonkwo et al., 2024). Having Black researchers and team members who belong to diverse vulnerable communities may facilitate the development of partnerships with predominantly Black and Brown communities, including community health agents, local community centers, and churches. Building trust-based relationships with these communities has been identified as the most effective strategy for recruiting African Americans into Alzheimer’s disease and dementia studies (Zhou et al., 2017; Lazaar et al., 2025).

The effectiveness of community-based trust-building strategies is supported by longstanding barriers related to mistrust in research practices and limited understanding of research procedures. For example, one study found that African American participants expressed fears of being used as “guinea pigs” and believed that research institutions “prey on vulnerable populations” (Shaw et al., 2023). These concerns are historically grounded, given the well-documented atrocities committed in clinical research involving African descendant populations during the past century, including the tragic Tuskegee Syphilis Study (Thomas and Quinn, 1991). Consequently, passive recruitment strategies, such as email invitations, flyers, and newspaper advertisements, tend to be less effective for socioeconomically disadvantaged populations (Lazaar et al., 2025).

A diverse research team contributes to the development of culturally appropriate digital recruitment campaigns. Cultural inadequacies in existing digital strategies may partially explain their limited effectiveness in reaching Black participants (Okonkwo et al., 2024). A protocol developed by the ADNI3 Diversity Taskforce (Okonkwo et al., 2024) combined centralized and local community engagement with digital campaigns designed and tested by African American and Latinx communities in the United States. These coordinated efforts substantially increased the recruitment of Black and Latinx participants. In addition, digital campaigns should also be targeted through social media algorithms to effectively reach Black populations.

This study presents certain limitations. As a narrative review, there is an inherent risk that some relevant publications on dementia, cognitive decline, and cognitive aging among Black Brazilians may not have been captured, which could partially constrain our inferences and recommendations. Nonetheless, comprehensive searches were conducted in major databases, followed by a meticulous, article-by-article analysis of the identified studies. These procedures provided a robust overview of the topic and yielded valuable insights. As a strength, no narrative review addressing this specific topic was identified in the databases searched. The only narrative review retrieved focused on Black Brazilians residing in the United States, underscoring the originality and relevance of the present study in the Brazilian context.

In conclusion, the literature on dementia, cognitive decline, and cognitive aging among the Brazilian Black population highlights a strong influence of socioeconomic factors on cognitive decline and dementia development. This finding suggests a significant potential for prevention and underscores the need for public policies that address social, income, and educational inequities affecting Black Brazilians. Beyond social investments, it is essential to prioritize local research aimed at developing culturally appropriate tools for assessing cognitive performance among marginalized populations across different regions of the country. Such efforts are critical for advancing understanding of cognitive decline and dementia in diverse Brazilian populations, including Black individuals, and for generating high-quality evidence through cohorts and clinical trials. Research should explore culturally compatible activities and lifestyles that serve as protective factors against dementia. Additionally, there is an urgent need to design culturally tailored strategies for care assessment and person-centered care for individuals living with dementia, as well as strategies to support family members and reduce their burden. Ensuring the inclusion of Black populations in future research will require investment in recruiting Black researchers and professionals into research teams, conducting research interventions within Black and Brown communities, and developing sustainable community partnerships.
