# Supplementary material for: Dementia and cognitive decline in Black Brazilians: a narrative review
Source: Front Hum Neurosci. 2026 Apr 22;20:1788136. doi: 10.3389/fnhum.2026.1788136 (PMC13143976; doi:10.3389/fnhum.2026.1788136)
Supplement: Supplementary file 1 [file Data_Sheet_1.docx]

| **SEARCH STRATEGIES** | | | |  |  | |  |
| --- | --- | --- | --- | --- | --- | --- | --- |
|  | | | |  |  | |  |
| DATABASE | |  | | |  | | |
| **Pubmed and Scielo** | | | | | | | |
|  | **Query 1**  (Dementia [MeSH] OR "cognitive decline" OR "cognitive aging" [MeSH] OR "cognitive impairment" OR Alzheimer Disease [MeSH] OR cognitive decline [Title/Abstract] OR dementia [Title/Abstract] OR cognitive aging [Title/Abstract] OR cognitive impairment [Title/Abstract] OR Alzheimer [Title/Abstract]) ***AND*** ("Brazil" [MeSH] OR "Brazil" [Affiliation] OR "Brazil" [Title/Abstract] OR "Brazilian" [Title/Abstract]) ***AND*** ("Black People" [MeSH] OR Black Person [MeSH] OR Negroid Race [MeSH] OR "African Continental Ancestry Group" [Title/Abstract] OR "Blacks" [Title/Abstract] OR "Black People" [Title/Abstract] OR Black Person [Title/Abstract] OR "black" [Title/Abstract] OR brown [Title/Abstract] OR "Afro-Brazilian" [Title/Abstract] OR "racial disparities" [Title/Abstract]) | | | | | | |
|  | **Query 2**  (Dementia [MeSH] OR cognitive decline OR cognitive aging [MeSH] OR cognitive impairment OR Alzheimer Disease [MeSH] OR cognitive decline [Title/Abstract] OR dementia [Title/Abstract] OR cognitive aging [Title/Abstract] OR cognitive impairment [Title/Abstract] OR Alzheimer [Title/Abstract]) ***AND*** ("Brazil" [MeSH] OR "Brazil" [Affiliation] OR "Brazil" [Title/Abstract] OR "Brazilian" [Title/Abstract]) ***AND*** ("Black People" [MeSH] OR Black Person [MeSH] OR Negroid Race [MeSH] OR "African Continental Ancestry Group" [Title/Abstract] OR "Blacks" [Title/Abstract] OR "Black People" [Title/Abstract] OR Black Person [Title/Abstract] OR "black" [Title/Abstract] OR brown [Title/Abstract] OR "Afro-Brazilian" [Title/Abstract] OR "racial disparities" [Title/Abstract]) ***AND*** ("social determinants of health" [MeSH] OR "social vulnerability" [MeSH] OR "health equity" [MeSH] OR "Racism" [MeSH] OR "social determinants of health" [Title/Abstract] OR "social vulnerability" [Title/Abstract] OR "health equity" [Title/Abstract] OR "Racism" [Title/Abstract] OR "Discrimination, Racial" [Title/Abstract] OR "Social Inequalities" [Title/Abstract] OR "discrimination" [Title/Abstract]) | | | | | | |
| **Lilacs** | | |  | | | | |
|  | **Query 1**  (mh:(Demência)) OR (mh:(Declínio Cognitivo)) OR (mh:(Envelhecimento Cognitivo)) OR (mh:(Comprometimento Cognitivo)) OR (mh:(Doença de Alzheimer)) OR (declínio cognitivo) OR (Demência) OR (envelhecimento cognitivo) OR (comprometimento cognitivo) OR (Alzheimer) ***AND*** (mh:(Brasil)) OR (Brasil) OR (Brasileiro) ***AND*** (mh:(Grupo de Ancestrais do Continente Africano)) OR (mh:(Pessoas Negras)) OR (mh:(População Negra)) OR (Afrodescendente) OR (Afrodescendentes) OR (negro) OR (preto) OR (pardo) OR (afro-brasileiro) OR (disparidades raciais) OR (População Negra) OR (Afrodescendente) OR (Grupo de Ancestrais do Continente Africano) OR (Pessoas Negras) | | | | | | |
|  | **Query 2**  (mh:(Demência)) OR (mh:(Declínio Cognitivo)) OR (mh:(Envelhecimento Cognitivo)) OR (mh:(Comprometimento Cognitivo)) OR (mh:(Doença de Alzheimer)) OR (declínio cognitivo) OR (Demência) OR (envelhecimento cognitivo) OR (comprometimento cognitivo) OR (Alzheimer) ***AND*** (mh:(Brasil)) OR (Brasil) OR (Brasileiro) ***AND*** (mh:(Grupo de Ancestrais do Continente Africano)) OR (mh:(Pessoas Negras)) OR (mh:(População Negra)) OR (Afrodescendente) OR (Afrodescendentes) OR (negro) OR (preto) OR (pardo) OR (afro-brasileiro) OR (disparidades raciais) OR (População Negra) OR (Afrodescendente) OR (Grupo de Ancestrais do Continente Africano) OR (Pessoas Negras) ***AND*** (mh:(Determinantes Sociais da Saúde)) OR (mh:(Vulnerabilidade Social)) OR (Determinantes Sociais da Saúde) OR (Vulnerabilidade Social) OR (mh:(Equidade em Saúde)) OR (mh:(Racismo)) OR (Racismo) OR (Equidade em Saúde) OR (mh:(Discriminação Racial)) OR (Discriminação Racial) OR (mh:(Desigualdades Sociais)) OR (mh:(Desigualdades Sociais)) OR (discriminação) | | | | | | |
| ARTIFICIAL INTELLIGENCE-BASED TOOL | | | | | |  | |
| **Scispace** | | | | | | | |
|  | | | **Query 1**  (Dementia [MeSH] OR "cognitive decline" OR "cognitive aging" [MeSH] OR "cognitive impairment" OR Alzheimer Disease [MeSH] OR cognitive decline [Title/Abstract] OR dementia [Title/Abstract] OR cognitive aging [Title/Abstract] OR cognitive impairment [Title/Abstract] OR Alzheimer [Title/Abstract]) ***AND*** ("Brazil" [MeSH] OR "Brazil" [Affiliation] OR "Brazil" [Title/Abstract] OR "Brazilian" [Title/Abstract]) ***AND*** ("Black People" [MeSH] OR Black Person [MeSH] OR Negroid Race [MeSH] OR "African Continental Ancestry Group" [Title/Abstract] OR "Blacks" [Title/Abstract] OR "Black People" [Title/Abstract] OR Black Person [Title/Abstract] OR "black" [Title/Abstract] OR brown [Title/Abstract] OR "Afro-Brazilian" [Title/Abstract] OR "racial disparities" [Title/Abstract]) | | | | |
|  | | | **Query 2**  (Dementia [MeSH] OR cognitive decline OR cognitive aging [MeSH] OR cognitive impairment OR Alzheimer Disease [MeSH] OR cognitive decline [Title/Abstract] OR dementia [Title/Abstract] OR cognitive aging [Title/Abstract] OR cognitive impairment [Title/Abstract] OR Alzheimer [Title/Abstract]) ***AND*** ("Brazil" [MeSH] OR "Brazil" [Affiliation] OR "Brazil" [Title/Abstract] OR "Brazilian" [Title/Abstract]) ***AND*** ("Black People" [MeSH] OR Black Person [MeSH] OR Negroid Race [MeSH] OR "African Continental Ancestry Group" [Title/Abstract] OR "Blacks" [Title/Abstract] OR "Black People" [Title/Abstract] OR Black Person [Title/Abstract] OR "black" [Title/Abstract] OR brown [Title/Abstract] OR "Afro-Brazilian" [Title/Abstract] OR "racial disparities" [Title/Abstract]) ***AND*** ("social determinants of health" [MeSH] OR "social vulnerability" [MeSH] OR "health equity" [MeSH] OR "Racism" [MeSH] OR "social determinants of health" [Title/Abstract] OR "social vulnerability" [Title/Abstract] OR "health equity" [Title/Abstract] OR "Racism" [Title/Abstract] OR "Discrimination, Racial" [Title/Abstract] OR "Social Inequalities" [Title/Abstract] OR "discrimination" [Title/Abstract]) | | | | |
